# Genome editing for horticultural crop improvement

**DOI:** 10.1038/s41438-019-0196-5

**Published:** 2019-10-08

**Authors:** Jiemeng Xu, Kai Hua, Zhaobo Lang

**Affiliations:** Shanghai Center for Plant Stress Biology, National Key Laboratory of Plant Molecular Genetics, Center of Excellence in Molecular Plant Sciences, Chinese Academy of Sciences, Shanghai, 200032 China

**Keywords:** Agricultural genetics, Transgenic plants

## Abstract

Horticultural crops provide humans with many valuable products. The improvement of the yield and quality of horticultural crops has been receiving increasing research attention. Given the development and advantages of genome-editing technologies, research that uses genome editing to improve horticultural crops has substantially increased in recent years. Here, we briefly review the different genome-editing systems used in horticultural research with a focus on clustered regularly interspaced palindromic repeats (CRISPR)/CRISPR-associated 9 (Cas9)-mediated genome editing. We also summarize recent progress in the application of genome editing for horticultural crop improvement. The combination of rapidly advancing genome-editing technology with breeding will greatly increase horticultural crop production and quality.

## Introduction

As an important branch of agriculture, horticulture originated thousands of years ago and has developed greatly during the course of human history. Horticultural crops are generally considered to include vegetable and fruit crops as well as floricultural and ornamental plants, which are cultivated for food, for nutritional and medical use, and for esthetic enjoyment^1^. Vegetable and fruit crops are low in calories but contain high levels of vitamins and minerals^2^, making them indispensable for balancing our daily diet. Although the supply of horticultural products is increasing, the diversity and nutritional value of the products are decreasing^3^. These decreases can be partially attributed to the narrow genetic diversity of horticultural crops resulting from domestication and breeding as well as reproductive barriers that inhibit genetic introgression from wild relatives. Therefore, the generation of genetic resources with diverse and desirable characteristics will be of great value for improving horticultural products.

Thousands of years ago, humans began to improve crops by introducing new traits from crossable relatives. The essential goal of this process was the transfer of desirable genetic variations. As late as 1930s, the available variations were generated solely through natural or spontaneous processes. Breeders subsequently learned to produce mutants by using chemical mutagens or radiation^4^. Both spontaneous and induced mutations have significantly increased crop yield and quality^5^. Given the rareness and randomness of these mutations, however, obtaining suitable materials for crop improvement has proven to be laborious and time consuming^4^.

With the rapid progress in molecular biology, DNA sequence-specific manipulation has become a powerful tool. In 1987, several animal scientists invented gene-targeting technology that relies on homologous recombination (HR). This innovative technology enabled researchers to precisely edit (though with a low frequency) an endogenous gene after introducing a donor template into mouse embryonic stem cells^6,7^. Similar progress was subsequently reported by plant researchers, but with an extremely low editing frequency of 0.5–7.2 × 10^−4 ^^8,9^. DNA double-stranded breaks (DSBs), which commonly result in HR in meiotic chromosomes^10^, were later used to increase the HR frequency in gene targeting^11^. In addition to HR, DSBs can be repaired through the error-prone nonhomologous end-joining (NHEJ) pathway in somatic cells, which can generate mutations via the small deletions or insertions that occur at a break site^12^. Scientists have used the following kinds of engineered endonucleases to introduce site-specific DSBs: meganucleases (MNs), zinc finger nucleases (ZFNs), transcription activator-like effector nucleases (TALENs), clustered regularly interspaced short palindromic repeats (CRISPR)/CRISPR-associated 9 (Cas9), and CRISPR from *Prevotella* and *Francisella* 1 (CRISPR/Cpf1). These engineered endonucleases have enabled genome editing in various biological systems^13–16^.

With the advent of CRISPR/Cas9, the application of genome editing to horticultural crops has greatly advanced. In this review, we first introduce and compare the engineered nucleases that are used for genome editing. We then consider their current applications in horticulture. Finally, we discuss the implications and challenges of genome editing for the improvement of horticultural crops.

## Genome-editing systems

Sequence-specific DNA binding, such as the interaction between a transcription factor and a promoter, is a common phenomenon. For genome editing, the previously mentioned nucleases can target specific sequences to generate DSBs under the guidance of protein–DNA interaction (for MNs, ZFNs, and TALENs) or RNA–DNA base-pairing (for CRISPR/Cas9 and CRISPR/Cpf1)^16,17^.

### Meganucleases or homing nucleases

**Fig. 1:**
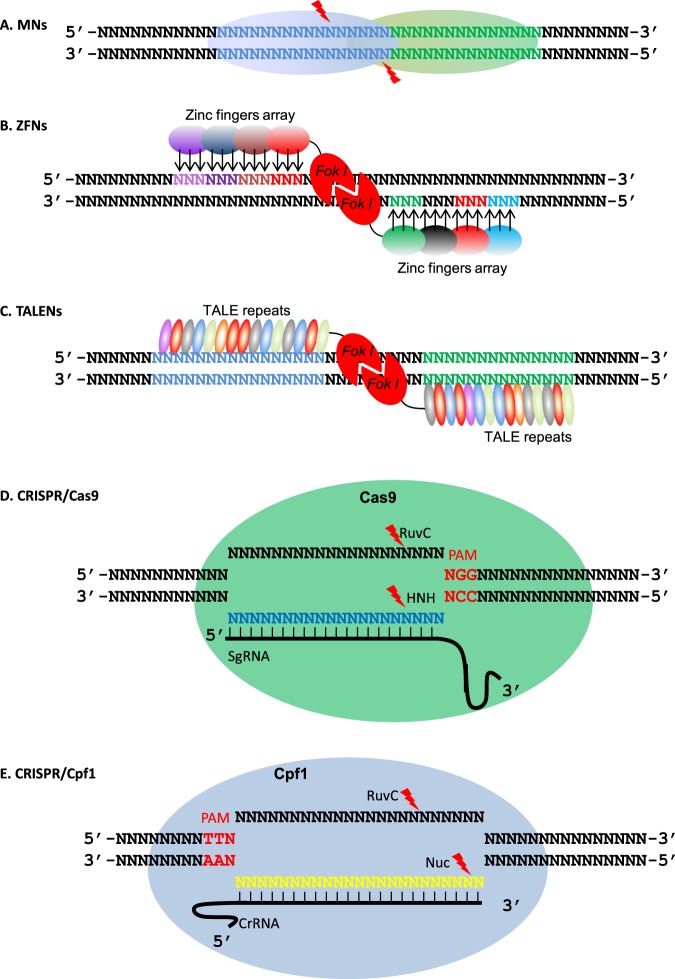
Schematic models of genome-editing systems. **a** A meganuclease can recognize a DNA sequence element of 12–40 bp and cut both strands at specific sites, forming sticky double-stranded breaks (DSBs). **b** In ZFNs, each zinc finger recognizes a 3-bp DNA sequence. Target specificity is achieved by arrays of several zinc fingers. Each DNA strand is bound by one zinc finger array linked with FokI, which in dimer form cuts DNA strands. **c** In TALENs, the central binding domain of each TALE consists of 13–28 repeats. Each repeat (a highly conserved sequence of 34 amino acids) can recognize and bind one nucleotide through the variable di-residues at the 12th and 13th positions. Paired TALENs lead to the dimerization of FokI, and the dimers cut the DNA stands, forming sticky DSBs at the target site. **d** In the CRISPR/Cas9 system, a single guide RNA (sgRNA) pairs with the target sequence upstream of a 5′-NGG-3′ PAM motif (N=A, T, C or G). The Cas9 endonuclease cuts the noncomplementary and complementary DNA strands at a location 3 nucleotides upstream of the PAM motif with RuvC and HNH domains, respectively. The cutting forms a blunt end DSB. **e** In the CRISPR/Cpf1 system, target specificity is achieved by the pairing of crRNA with the DNA strand downstream of a 5′-TTN-3′ PAM motif. The Cpf1 endonuclease uses the RuvC and Nuc domains to cut noncomplementary and complementary DNA strands at different positions, producing DSBs with sticky ends

The first class of nucleases for genome editing, MNs or homing endonucleases, was discovered in the genomes of microorganisms or organelles. By recognizing DNA sequence elements ranging from 12 to 40 bp, these nucleases cut both strands of DNA in a site-specific manner (Fig. 1a)^18^. Among MNs, the I-*Cre*I protein has received the most research attention and has been reported to be effective in maize^19^, but the rare occurrence of recognizable sites limits the ability of I-*Cre*I and other MNs to edit desired target sites^17^. To broaden the application of MNs, researchers have used mutagenesis or combinatorial assembly to produce MN variants that target the desired DNA sequence^20,21^. Nevertheless, the overlapping recognition and catalytic domains of modified MNs cause difficulties and often compromise their catalytic activity^15^. For these reasons, MNs have not been widely used by plant scientists.

### ZFNs and TALENs

As suggested by their names, ZFNs or TALENs are generated by fusing the DNA cleavage domain of the endonuclease FokI with zinc fingers (ZFs) or with transcriptional activator-like effectors (TALEs). The FokI endonuclease domain mediates independent and nonspecific DNA cleavage upon dimerization and is not involved in any sequence recognition^22^. Therefore, a pair of ZFs or TALEs, each fused with a FokI endonuclease domain, is designed to achieve site-specific cleavage^23–25^. ZFs are found in transcription factors, with each finger domain recognizing three specific nucleotides. ZFNs typically exhibit an array of 3 or 4 finger domains, which can recognize 18–24 bp sequences when a ZFN occurs as a dimer^23,25^. Many studies have been conducted to improve ZFN applicability, efficiency, and precision^26,27^, but there are still concerns about interference from neighboring finger domains and the limited number of recognition sites (Fig. 1b)^15^.

In contrast to ZFNs, TALENs achieve sequence specificity via the customizable DNA-binding domains of TALEs, which are proteins excreted by the common bacterial plant pathogen *Xanthomonas*^28^. During pathogenesis, TALEs bind to a specific sequence of plant promoters to activate gene expression to facilitate infection^28^. The central binding domain of TALEs consists of 13–28 repeat sequences. Each repeat, which encodes a highly conserved sequence of 34 amino acids, can recognize and bind to one nucleotide through the variable di-residues at the 12th and 13th positions^29–31^. Such one-to-one pairing, together with the negligible context dependency on neighboring repeats, enables TALENs to target desired sequences (Fig. 1c)^32,33^. In general, TALENs outperform ZFNs in terms of precision and accessibility.

### CRISPR/Cas9 and CRISPR/Cpf1

Unlike ZFN and TALEN systems, which depend on protein–DNA binding specificity, the CRISPR system relies on RNA–DNA binding to achieve sequence specificity. During the functional elucidation of the CRISPR/Cas system, its involvement in bacterial resistance to viruses was experimentally demonstrated^34^, and several components, including crRNA, PAM motif, and tracrRNA, were discovered to be necessary for this system^35–37^. More interestingly, reconstructed key components of the CRISPR/Cas9 system can introduce DSBs in a site-specific way, suggesting the potential use of this programmable RNA-guided CRISPR/Cas9 system for genome editing in organisms other than bacteria^38,39^. This possibility was soon demonstrated in human and mouse cells^40–42^, zebrafish^43^, and plants^44–48^. In the system, site-specific binding to the target is achieved via RNA-DNA pairing of a 20-nt sequence in the chimeric single-guide RNA (sgRNA) with the target. The other crRNA- and tracrRNA-derived sequences also interact with the target to form an RNA:DNA heteroduplex that is recognized by the collective interactions of several Cas9 domains: PI, REC1, RuvC, and NUC. Thereafter, the RuvC and HNH domains cut the noncomplementary and complementary DNA strands at a location 3 nucleotides upstream of the PAM motif, respectively (Fig. 1d). The recognizable PAM motif of Cas9 is 5′-NGG-3′ (N=A, T, C, or G), and this G-rich feature prevents the design of sgRNAs in T-rich regions^49^.

Cpf1, another endonuclease in the class 2 Type V CRISPR system, has also been found to be efficient in plant genome editing^50^ and to present unique features^51^. First, Cpf1 does not require an additional tracrRNA to form a mature crRNA. Second, unlike Cas9, which recognizes G-rich PAM sequences, Cpf1 recognizes T-rich PAM sequences. Finally, whereas cutting by the Cas9 endonuclease produces blunt ends, cutting by the Cpf1 endonuclease produces cohesive ends (Fig. 1e). In addition to causing site-specific mutations, CRISPR genome-editing systems can be used to achieve gene regulation^52,53^ through the manipulation of the nuclease-inactivated Cas9 (dCas9).

Each of the endonucleases used for genome editing has unique properties because of differences in their underlying mechanisms (Fig. 1 and Table 1, Zhang et al.^16,54^; Knott and Doudna^55^). In addition to generating indel mutations at target sequences, CRISPR/Cas systems have been adapted for precise base editing^56–59^. Base editors usually consist of an sgRNA-guided Cas9 nickase (nCas9) fused with a deaminase that causes C to T or A to G base conversions. These resources greatly increase the versatility of the tools that can be used for precise manipulation of horticultural crops.

**Table 1:** Comparison of genome-editing systems*

Property	MNs	ZFNs	TALENs	CRISPR/Cas9 or CRISPR/Cpf1
Site-recognition domain	MN binding domain	Zinc fingers	Transcription activator-like effectors	sgRNA or crRNA
Interaction pattern	Protein–DNA	Protein–DNA	Protein–DNA	RNA–DNA pairing
DNA cleavage	MNs	FokI	FokI	Cas9 or Cpf1
Available sites**	1/1000 bp	1/140 bp	Any site (in principle)	1/13 bp
Precision	+	++	++++	+++ or ++++
Efficiency	+	+	++	++ or ++
Ease of design	+	++	+++	++++ or +++++
Specificity	++	++	+++	+ or +++
Multiplex editing	+	+	++	+++ or ++++

*This table is based on Boglioli and Richard^60^, Rocha-Martins et al.^17^, and Zhang et al.^16^. “+” indicates the level

**This information is based on human genome data

## Current status of genome editing in horticultural crops

**Fig. 2:**
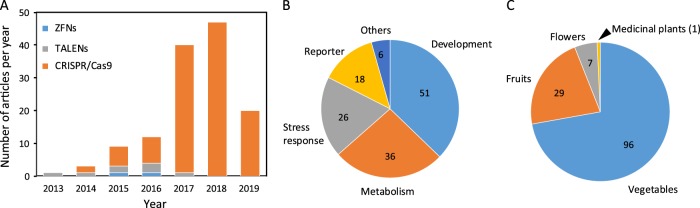
Number of research articles involving gene editing. The information used in this figure was retrieved through May 31 of 2019. According to the information from https://aps.dac.gov.in/Public/Crops.pdf, horticultural crops include vegetables, fruits, florals, and medicinal plants. **a** The number of research articles involving the editing of horticultural crops with ZFNs, TALENs, and CRISPR/Cas9 from 2013 to 2019 (only the first 5 months). **b** The number of research articles in which the edited genes were mainly associated with development, metabolism, stress tolerance and other functions. **c** The number of research articles involving gene editing of different kinds of horticultural crops

To obtain genetic resources with diverse characteristics for breeding, both spontaneous and induced mutations have been commonly used^60^. The rareness and uncertainty of these mutations have motivated scientists to find ways to introduce precise mutations at target sites^15,17^. Recently, most genome-editing studies on plants have been carried out in model systems and staple crops^44–46^, but the application of genome editing to horticultural crops is rapidly increasing^61^. In 2013, the first example of genome editing in a horticultural crop was achieved via a TALEN in *Brassica* *oleracea*^62^. In the following years, the number of studies involving genome editing in horticulture has exponentially increased (Fig. 2a, Table 2), and CRISPR-based systems now dominate. The functions of genes targeted by genome editing are very diverse, but researchers have focused most on targets affecting development, followed by targets affecting metabolism and stress responses. In addition, studies that focus on the improvement of the CRISPR/Cas9 system in horticultural crops frequently use marker/reporter genes as targets such as *phytoene desaturase* (*PDS*), whose mutation results in an albino phenotype (Fig. 2b). Among horticultural crops, tomato has received much more attention regarding genome editing than other crops: ~42% of genome-editing studies have involved tomato, whereas ~13% have involved potato. Although most (72%) genome editing with horticultural crops is performed in vegetables (Fig. 2c), some floral and medicinal plants have also been successfully manipulated by genome editing (Fig. 2c).

**Table 2:** A list of publications on genome editing in horticultural crops

Species	Crop type	Genome editing tool	Targeted gene	Gene function or phenotype	Classification of targeted gene	Reference
*Solanum lycopersicum*	Vegetable	CRISPR	*SlALS1*	Enhanced herbicide resistance	Stress response	^103^
*Solanum lycopersicum*	Vegetable	CRISPR	*SlJAZ2*	Resistance to bacterial speck	Stress response	^104^
*Solanum lycopersicum*	Vegetable	CRISPR	*APETALA2a (AP2a), NON-RIPENING (NOR) and FRUITFULL (FUL1/TDR4 and FUL2/MBP7)*	Fruit development and ripening	Development	^105^
*Solanum lycopersicum*	Vegetable	CRISPR	*Pectate lyase (PL), polygalacturonase 2a (PG2a), and beta-galactanase (TBG4)*	Cell wall gene, altered fruit color and firmness	Development	^106^
Solanum lycopersicum	Vegetable	CRISPR	*SlNPR1*	Reduced drought tolerance	Stress response	^107^
*Solanum lycopersicum*	Vegetable	CRISPR	*SlALS1, SlALS2*	Enhanced herbicide resistance	Stress response	^101^
*Solanum lycopersicum*	Vegetable	CRISPR	*SlGAI*	Gibberellin response and dwarfism	Development	^108^
*Solanum lycopersicum*	Vegetable	CRISPR	*SlEIN2, SlERFE1, SlARF2B, SlGRAS8, SlACS2, SlACS4*	Ethylene response and fruit development	Development	^97^
*Solanum lycopersicum*	Vegetable	CRISPR	*SBPase*	Leaf senescence (SBPase in primary metabolism)	Metabolism	^109^
*Solanum lycopersicum*	Vegetable	CRISPR	*CBF1*	Chilling tolerance	Stress response	^110^
*Solanum lycopersicum*	Vegetable	CRISPR	*POLYGALACTURONASE (PG) and PECTATE LYASE (PL)*	Cell wall gene	Development	^111^
*Solanum lycopersicum*	Vegetable	CRISPR	*NPTII*	N.A.	Others	^112^
*Solanum lycopersicum*	Vegetable	CRISPR	*Psy1 and CrtR-b2*	Carotenoid metabolism	Metabolism	^113^
*Solanum lycopersicum*	Vegetable	CRISPR	*NADK2A, IAA9*	NAD Kinase 2A; IAA9	Development	^114^
*Solanum lycopersicum*	Vegetable	CRISPR	*DDM1a, b*	Decrease in DNA methylation	Development	^115^
*Solanum lycopersicum*	Vegetable	CRISPR	*SlMAPK20*	Aborted pollen development	Development	^116^
*Solanum lycopersicum*	Vegetable	CRISPR	*Carotenoid isomerase and Psy1*	Carotenoid metabolism	Metabolism	^117^
*Solanum lycopersicum*	Vegetable	CRISPR	*Solyc08g075770*	Fusarium wilt susceptibility	Stress response	^118^
*Solanum lycopersicum*	Vegetable	CRISPR	*TypeII GRX 14, 15, 16, 17*	Redox regulation	Metabolism	^119^
*Solanum lycopersicum*	Vegetable	CRISPR	*lncRNA1459*	Repressed fruit ripening, lycopene, ethylene and carotenoid biosynthesis	Metabolism	^120^
*Solanum lycopersicum*	Vegetable	CRISPR	*SGR1, Blc, LCY-E, LCY-B1, LCY-B2*	Increased lycopene content	Metabolism	^121^
*Solanum lycopersicum*	Vegetable	CRISPR	*PDS*	Albino phenotype	Reporter	^122^
*Solanum lycopersicum*	Vegetable	CRISPR	*SlDML2*	DNA methylation and fruit ripening	Reporter	^66^
*Solanum lycopersicum*	Vegetable	CRISPR	*PDS and GABA-TP1, GABA-TP2, GABA-TP3, CAT9 and SSADH*	γ-aminobutyric acid metabolism	Metabolism	^123^
*Solanum lycopersicum*	Vegetable	CRISPR	*SlMYB12*	Pink tomato fruit color	Metabolism	^124^
*Solanum lycopersicum*	Vegetable	CRISPR	*Coat protein, Replicase from TYLCV*	Obtained resistance to tomato yellow leaf curl virus	Stress response	^125^
*Solanum lycopersicum*	Vegetable	CRISPR	*RIN*	Ethylene production and fruit ripening	Metabolism	^126^
*Solanum pimpinellifolium*	Vegetable	CRISPR	*SP, MULT, FAS, CyCb, OVUTE and FW2.2*	Plant and inflorescence architecture, fruit shape and lycopene biosynthesis	Development, metabolism	^69^
*Solanum pimpinellifolium*	Vegetable	CRISPR	*SP, SP5, CLV3 and WUS, GGP1*	plant architecture, day-length insensitivity, enlarged fruit size and vitamin C	Development, metabolism	^70^
*Solanum lycopersicum*	Vegetable	CRISPR	*RIN*	Ethylene production and fruit ripening	Development	^68^
*Solanum lycopersicum*	Vegetable	CRISPR	*SlORRM4*	RNA editing and fruit ripening	Development	^67^
*Solanum lycopersicum*	Vegetable	CRISPR	*ALC*	Shelf life	Metabolism	^127^
*Solanum lycopersicum*	Vegetable	CRISPR	*CLAVATA-WUSCHEL*	Altered locule number	Development	^65^
*Solanum lycopersicum*	Vegetable	CRISPR	*SlMAPK3*	Drought stress	Stress response	^128^
*Solanum lycopersicum*	Vegetable	CRISPR	*Glutamate decarboxylase (GAD)*	γ-aminobutyric acid metabolism	Metabolism	^129^
*Solanum lycopersicum*	Vegetable	CRISPR	*Solyc12g038510*	Jointless mutant, abscission	Development	^130^
*Solanum lycopersicum*	Vegetable	CRISPR	*Multiple genes*	Generate a pool of mutants	Others	^131^
*Solanum lycopersicum*	Vegetable	CRISPR	*PSY*	Fruit color	Development	^132^
*Solanum lycopersicum*	Vegetable	CRISPR	*Solyc12g038510*	Jointless and branching	Development	^133^
*Solanum lycopersicum*	Vegetable	CRISPR	*L1L4*	Involved in fruit metabolism during ripening	Metabolism	^134^
*Solanum lycopersicum*	Vegetable	CRISPR	*DELLA and ETR*	Hormone response	Development	^135^
*Solanum lycopersicum*	Vegetable	CRISPR	*SlMlo1*	Powdery mildew resistance	Stress response	^136^
*Solanum lycopersicum*	Vegetable	CRISPR	*SlIAA9*	Parthenocarpic tomato plants	Development	^137^
*Solanum lycopersicum*	Vegetable	CRISPR	*SP5G*	More rapid flowering	Development	^64^
*Solanum lycopersicum*	Vegetable	CRISPR	*Genes involved tomato domestication*	Development and plant architecture	Development	^138^
*Solanum lycopersicum*	Vegetable	CRISPR	*SlAGL6*	Production of parthenocarpic fruit under high temperature	Development	^139^
*Solanum lycopersicum*	Vegetable	CRISPR	*N.A*	N.A	Others	^140^
*Solanum lycopersicum*	Vegetable	CRISPR	*SlBOP*	Inflorescence structure	Development	^63^
*Solanum lycopersicum*	Vegetable	ZFN	*L1L4*	Heterochronic phenotype, plant architecture	Development	^141^
*Solanum lycopersicum*	Vegetable	CRISPR	*PDS and PIF*	Albino phenotype	Reporter	^142^
*Solanum lycopersicum*	Vegetable	CRISPR	*N.A*.	N.A.	Others	^143^
*Solanum lycopersicum*	Vegetable	TALEN, CRISPR	*ANT1*	Anthocyanin biosynthesis	Metabolism	^144^
*Solanum lycopersicum*	Vegetable	CRISPR	*RIN*	Fruit ripening	Development	^145^
*Solanum lycopersicum*	Vegetable	TALEN	*PROCERA*	GA response and taller plant	Development	^146^
*Solanum lycopersicum*	Vegetable	CRISPR	*AGO7*	Leaf morphology	Development	^147^
*Solanum* *tuberosum*	Vegetable	CRISPR	*St16DOX*	Steroidal glycoalkaloids metabolism	Metabolism	^148^
*Solanum* *tuberosum*	Vegetable	CRISPR	*GBSS genes*	Starch biosynthesis	Metabolism	^149^
*Solanum* *tuberosum*	Vegetable	CRISPR	*S-RNase*	Self-incompatibility	Development	^150^
*Solanum* *tuberosum*	Vegetable	CRISPR	*Coilin gene*	Enhanced resistance to biotic and abiotic agents	Stress response	^151^
*Solanum* *tuberosum*	Vegetable	CRISPR	*StALS1, StALS2*	Enhanced herbicide resistance	Stress response	^101^
*Solanum* *tuberosum*	Vegetable	CRISPR	*GBSS1*	Starch biosynthesis	Metabolism	^152^
*Solanum* *tuberosum*	Vegetable	CRISPR	*S-Rnase*	Self-incompatibility	Development	^72^
*Solanum* *tuberosum*	Vegetable	CRISPR	*Coilin gene*	Enhanced resistance to biotic and abiotic agents	Stress response	^153^
*Solanum* *tuberosum*	Vegetable	TALEN	*SBE1 and StvacINV22*	Sugar metabolism	Metabolism	^154^
*Solanum* *tuberosum*	Vegetable	CRISPR	*StMYB44*	Phosphorus homeostasis	Stress response	^155^
*Solanum* *tuberosum*	Vegetable	CRISPR	*GBSS*	Starch metabolism and tuber quality	Metabolism	^156^
*Solanum* *tuberosum*	Vegetable	TALEN	*StALS1*	Enhanced herbicide resistance	Stress response	^157^
*Solanum* *tuberosum*	Vegetable	TALEN	*StALS1*	Enhanced herbicide resistance	Stress response	^158^
*Solanum* *tuberosum*	Vegetable	TALEN	*vINV*	Postharvest cold storage and processing	Metabolism	^71^
*Solanum* *tuberosum*	Vegetable	CRISPR	*StALS1*	Enhanced herbicide resistance	Metabolism	^159^
*Solanum* *tuberosum*	Vegetable	TALEN	*StALS1*	Enhanced herbicide resistance	Metabolism	^160^
*Solanum* *tuberosum*	Vegetable	CRISPR	*StIAA2*	Aux/IAA protein, shoot morphogenesis	Development	^161^
*Brassica oleracea*	Vegetable	CRISPR	*BolC.GA4.a*	GA response and dwarfism	Development	^162^
*Brassica oleracea*	Vegetable	CRISPR	*BoPDS, BoSRK3, BoMS1*	Albino phenotype, self-incompatibility, male sterility	Development	^163^
*Brassica napus*	Vegetable	CRISPR	*LMI1*	Leaf lobe development	Development	^164^
*Brassica oleracea, rapa*	Vegetable	CRISPR	*PDS and FRI*	Albino phenotype and flowering	Reporter, development	^165^
*Brassica napus*	Vegetable	CRISPR	*FAD2*	Fatty acid metabolism	Metabolism	^76^
*Brassica carinata*	Vegetable	CRISPR	*Fascilin-like arabinogalactan protein*	Regulation of root hairs under phosphorus stress	Development, stress response	^166^
*Brassica napus*	Vegetable	CRISPR	*WRKY11 and WRKY70*	Enhanced biotic resistance	Stress response	^167^
*Brassica napus*	Vegetable	CRISPR	*SDG8*	Histone lysine methyltransferase	Development	^168^
*Brassica napus*	Vegetable	CRISPR	*CLV3 and CLV1, CLV2*	Regulate multilocular seeds	Development	^169^
*Brassica rapa and napus*	Vegetable	CRISPR	*AP2a, AP2b*	Sepal to carpal modification	Development	^170^
*Brassica napus*	Vegetable	CRISPR	*BnaRGA, BnaDA1, BnaDA2, BnaFUL*	Multiple genes involved in plant development	Development	^171^
*Brassica carinata*	Vegetable	CRISPR	*Fascilin-like arabinogalactan protein*	Root hair development	Development	^172^
*Brassica napus*	Vegetable	CRISPR	*ALC*	Valve margin development, seed shattering	Development	^173^
*Brassica oleracea*	Vegetable	TALEN	*FRIGIDA*	Early flowering phenotype	Development	^62^
*Dendrobium officinale*	Flower	CRISPR	*C3H, C4H, 4CL, CCR, and IRX*	Lignocellulose biosynthesis	Metabolism	^174^
*Lettuce sativa*	Vegetable	CRISPR	*LsBIN2*	Impaired brassinosteroid response	Development	^83^
*Lettuce sativa*	Vegetable	CRISPR	*LsNCED4*	Thermo-inhibition of seed germination	Development	^175^
*Cucumis sativus*	Vegetable	CRISPR	*eIF4E*	Enhanced viral resistance	Stress response	^176^
*Cucumis sativus*	Vegetable	CRISPR	*CmWIP1*	Gynoecious phenotype	Development	^177^
*Musa balbisiana*	Fruit	CRISPR	*eBSV*	Control of virus pathogenesis	Stress response	^178^
*Musa acuminata*	Fruit	CRISPR	*PDS*	Albino phenotype	Reporter	^179^
*Musa acuminata*	Fruit	CRISPR	*PDS*	Albino phenotype	Reporter	^180^
*Actinidia deliciosa*	Fruit	CRISPR	*PDS*	Albino phenotype	Reporter	^181^
*Vitis vinifera*	Fruit	CRISPR	*VvPDS*	Albino phenotype	Reporter	^182^
*Vitis vinifera*	Fruit	CRISPR	*IdnDH*	Biosynthesis of tartaric acid	Metabolism	^183^
*Vitis vinifera*	Fruit	CRISPR	*VvWRKY52*	Increased the resistance to *Botrytis cinerea*	Stress response	^75^
*Vitis vinifera*	Fruit	CRISPR	*VvPDS*	Albino phenotype	Reporter	^184^
*Vitis vinifera*	Fruit	CRISPR	*MLO-7*	Powdery mildew resistance	Stress response	^185^
*Vitis vinifera*	Fruit	CRISPR	*IdnDH*	Biosynthesis of tartaric acid	Metabolism	^186^
*Citrus sinensis*	Fruit	CRISPR	*DMR6*	Huanglongbin resistance	Stress response	^187^
*Citrus sinensis*	Fruit	CRISPR	*PDS*	Albino phenotype	Reporter	^188^
*Citrus paradisi*	Fruit	CRISPR	*CsPDS, Cs2g12470 and Cs7g03360*	Albino phenotype	Reporter	^189^
*Citrus sinensis*	Fruit	CRISPR	*PDS*	Albino phenotype	Reporter	^190^
*Citrus sinensis*	Fruit	CRISPR	*CsLOB1*	Canker resistance	Stress response	^73^
*Citrus paradisi*	Fruit	CRISPR	*CsLOB1*	Canker resistance	Stress response	^74^
*Citrus sinensis*	Fruit	CRISPR	*CsPDS*	Albino phenotype	Reporter	^191^
*Chrysanthemum morifolium*	Flower	CRISPR	*CpYGFP*	Targeted editing of the YGFP reporter gene	Others	^192^
*Ipomoea nil*	Flower	CRISPR	*InDFR-B*	Anthocyanin biosynthesis and white flowers	Metabolism	^193^
*Ipomoea nil*	Flower	CRISPR	*InCCD4*	Altered petal color	Development	^194^
*Petunia inflata*	Flower	CRISPR	*PiSSK1*	Self-incompatibility	Development	^195^
*Petunia hybrid*	Flower	CRISPR	*PDS*	Albino phenotype	Reporter	^196^
*Citrullus lanatus*	Fruit	CRISPR	*ALS*	Increased herbicide resistance	Stress response	^197^
*Citrullus lanatus*	Fruit	CRISPR	*PDS*	Albino phenotype	Reporter	^198^
*Salvia miltiorrhiza*	Medicinal plant	CRISPR	*SmCPS1*	Tanshinone biosynthesis	Metabolism	^199^
*Camelina sativa*	Vegetable	CRISPR	*FAE1*	Reduced long-chain fatty acids	Metabolism	^77^
*Camelina sativa*	Vegetable	CRISPR	*CsDGAT1 or CsPDAT1*	Altered fatty acid composition and reduced oil content	Metabolism	^200^
*Camelina sativa*	Vegetable	CRISPR	*FAD2*	Reduced levels of polyunsaturated fatty acids	Metabolism	^78^
*Camelina sativa*	Vegetable	CRISPR	*FAD2*	Decreased polyunsaturated fatty acids	Metabolism	^79^
Malus pumila	Fruit	CRISPR	*PDS, TFL1.1*	Albino phenotype, early flowering	Development	^201^
*Malus* *pumila*	Fruit	CRISPR	*PDS*	Albino phenotype	Reporter	^183^
*Malus* *pumila*	Fruit	CRISPR	*PDS*	Albino phenotype	Reporter	^202^
*Malus* *pumila*	Fruit	CRISPR	*DIPM*	Blight resistance	Stress response	^185^
*Malus* *pumila*	Fruit	ZFN	*udiA*	Edited reporter gene	Others	^203^
*Pyrus communis*	Fruit	CRISPR	*TFL1.1*	Early flowering	Development	^201^
*Daucus carota*	Vegetable	CRISPR	*PDS, MYB113-like*	Albino phenotype	Reporter	^204^
*Daucus carota*	Vegetable	CRISPR	*F3H*	Altered anthocyanin biosynthesis	Metabolism	^205^
*Torenia fournieri*	Flower	CRISPR	*F3H*	Altered flower pigmentation	Metabolism	^206^
*Fragaria vesca*	Fruit	CRISPR	*FveTAA1, FveARF8*	Auxin signaling, plant development	Development	^207^
*Fragaria vesca, Fragaria x Ananassa*	Fruit	CRISPR	*FvMYB10, FvCHS*	Anthocyanin biosynthesis	Metabolism	^208^
*Fragaria x Ananassa*	Fruit	CRISPR	*FaTM6*	Anther development	Development	^209^
*Fragaria vesca, Fragaria x Ananassa*	Fruit	CRISPR	*PDS*	Albino phenotype	Reporter	^210, 211^

In tomato, development-related genes have been edited to manipulate flowering patterns and fruit development. The tomato *BLADE-ON-PETIOLE* (*BOP*) genes, which encode transcriptional cofactors, can regulate inflorescence structure, and knock-out of *SlBOP* genes by gene editing reduces the number of flowers per inflorescence^63^. CRISPR/Cas9-induced mutations in the flowering repressor *self-pruning 5G* lead to rapid flowering and early harvest^64^. In addition, editing of the cis-regulatory region of *SlCLV3*^65^ or the coding regions of *SlDML2*^66^, *SlORRM4*^67^ and the *RIN* locus^68^ alters fruit development and ripening. Interestingly, multiplex targeting of several genes that are important for tomato domestication was found to greatly alter the properties of the wild tomato relative *Solanum pimpinellifolium* such that the generated mutants were similar to cultivated tomato^69,70^. In potato, when the vacuolar invertase gene was disrupted by TALEN, the cold storage and processing of tubers were improved^71^. Another recent study in potato showed the possibility of overcoming self-incompatibility by editing the *S-RNase* gene, which would provide an alternative method of propagation through seeds^72^. In addition to tomato and potato, other horticultural crops have also been edited to obtain desirable traits. Genes related to resistance to plant pathogens such as *Xanthomonas citri*^73,74^ and *Botrytis cinerea*^75^ have been manipulated in citrus, apple, and grape. In oilseed crops, genes involved in fatty acid metabolism have been frequently targeted to improve oil quality^76–79^. The application of genome editing to improve crops is based on knowledge of the association between genes and their controlled traits. In the future, functional characterization of genes in different crops will help to identify valuable targets that could be edited for potential horticultural improvement, such as increased productivity, marketing quality, and nutritional value.

## Possible implications of genome editing in horticulture

The goal of breeding is to harness genetic variations to introduce desirable traits. These genetic variations can arise in various ways, such as by spontaneous mutation, chemical mutagenesis, and physical mutagenesis. Gene editing could be regarded as biological mutagenesis. In comparison with other approaches, genome-editing technology is superior in terms of versatility, efficiency, and specificity. For instance, CRISPR-based genome editing can cause many types of mutations in target sequences, including small insertions/deletions, deletions of large fragments, gene replacement, and precise base substitutions^16^. In addition, genome-editing technology is continuously advancing: the endonuclease Cpf1^51^ and newly discovered or designed Cas9 variants^80,81^ can recognize different PAM sequences, thereby broadening the genome-wide sites that can be targeted for editing.

Genome-edited plants are not considered genetically modified organisms (GMOs) in countries such as the U.S. and Japan but are still under strict GMO regulation in Europe. The largest difference between genome-edited plants and GMOs is that the genomes of edited plants can be free of exogenous DNA sequences. The exogenous DNA of the editing tools can be removed through genetic segregation^82^ or may never have to be introduced if CRISPR reagents are delivered as ribonucleoproteins^83,84^.

Mutants generated via genome editing can be directly used for crop production or as prebreeding materials. Through genome editing, desirable traits can be directly introgressed into elite or heirloom lines without compromising other properties, and the resulting lines with targeted improvement will be ready for use in production. The wild relatives of cultivated varieties are also potential materials for genome editing because they generally present unique features in many important traits. For instance, wild species of cultivated tomato are more resistant to unfavorable environments than commercial cultivars^85^. Wild *Solanum pimpinellifolium* was recently domesticated by the editing of several important genes affecting plant architecture and fruit development, resulting in new tomato varieties with the desirable properties of cultivated tomato combined with the favorable traits of the wild species^69,70^. Mutations can generally be introduced in either the coding region or the cis-regulatory region of the targeted gene, and mutations in the cis-regulatory region could be used to generate quantitative variation for breeding selection. In tomato, for example, fruit locule number is determined by several naturally occurring mutations in the cis-regulatory regions of *CLAVATA-WUSCHEL*^65^. This finding motivated researchers to design a multiplexed CRISPR/Cas9 system targeting the *CLAVATA-WUSCHEL* promoters to generate tomato lines with a wide range of locule numbers. Quantitative variations have also been observed when the genes responsible for inflorescence and plant architecture are engineered^65^. In addition to regulating gene activity by editing the DNA sequence of the cis-regulatory region, gene activity can be regulated by the its epigenetic status of this region. By integrating genome editing (CRISPR/Cas9) with epigenetic regulation, researchers are able to target a gene of interest and modify its epigenetic status. For instance, an sgRNA-guided fusion protein between the dead Cas9 (dCas9) variant and the catalytic domain of the TEN-ELEVEN TRANSLOCATION1 (TET1cd) demethylase can remove 5mC at specific sites, thereby increasing gene expression^86^. An epigenetic mutant can also be crossed with the corresponding wild type to generate epigenetic recombinant inbred lines (epiRILs). Individuals from these populations are genetically identical but epigenetically distinct. Such populations have been constructed in Arabidopsis and exhibit considerable phenotypic variations^87–90^. These examples demonstrate that genome editing is an excellent tool for producing new alleles and epialleles, which are important sources of phenotypic variation for crop improvement.

## Challenges and future perspectives for the improvement of horticultural crops through genome editing

Although genome editing has many advantages over conventional crop breeding, some challenges remain for its application to horticultural crops. In horticultural crops, molecular and genetic studies are difficult, which hinders the identification of genes responsible for desirable traits. Sequencing the genomes of horticultural crops of interest will be important for identifying genes associated with desirable traits. For crops lacking a reference genome, the target sequence could be cloned by using degenerate primers designed for conserved protein motifs with putative functions related to desirable traits. A good example is the *mildew-resistance locus* (*MLO*), which has been characterized in detail in barley^91^; the phylogenetically conservative nature of the *MLO* has facilitated the generation of powdery mildew-resistant plants in wheat, tomato, and strawberry^92,93^.

Once a gene to be edited has been identified, researchers must take into account the methods used to deliver editing reagents and the procedure for regenerating the edited mutants. To date, more than 25 horticultural plant species have been successfully edited (Table 2), usually with editing reagents delivered via *Agrobacteria* or virus systems, and the edited plants are regenerated via in vitro tissue culture. Although tissue culture-based transformation and regeneration is most widely used for genome editing, no well-established protocol for transformation and regeneration from tissue culture is available for many horticultural crops. *In planta* transformation, which is an alternative to in vitro tissue culture-based *Agrobacterium* transformation, refers to the infection of in vivo explants in which the targeted tissues are apical or auxiliary meristems, stigmas, pollens, or inflorescences^94^. This method has been successfully used to transform tomato^95^ and *Brassica* species^96^ and should be further explored for use in horticultural crops that are recalcitrant to traditional genetic transformation. Additionally, successful genetic transformation of horticultural crops requires the consideration of editing efficiency, which is affected by many factors, such as sgRNA number and GC content, the expression levels of sgRNA and Cas9, and the secondary structure of the paired sgRNA and target sequence^97,98^. In the future, the editing system should be further optimized in different crop species.

The elimination of foreign DNA fragments (transferred T-DNAs) to obtain transgene-free edited plants remains difficult in some highly heterozygous and clonally propagated horticultural species^99^, such as potato, sweet potato, and banana. One possibility is to generate many transformants, followed by high-throughput screening of transgene-free mutants^100^. This approach has been used to generate ~10% of mutants without foreign DNA^100,101^. Another approach for transgene-free genome editing is to deliver editing reagents as in vitro transcripts^102^ or ribonucleoproteins^83,84^.

In conclusion, mutagenesis via genome editing outperforms spontaneous and induced mutations in terms of precision and efficiency. Although this technology is being increasingly used in many crops, its widespread use in the breeding of horticultural crops will require three challenges to be surmounted. First, clear breeding traits of the horticultural crop in question should be identified via communication among consumers, breeders, and biologists. Second and third, suitable methods must be developed for delivering editing reagents and for subsequently regenerating mutants. Given the great potential of genome editing and the importance of horticultural crops, we expect that these challenges will be overcome in the near future.
